# Community engagement: health research through informing, consultation, involving and empowerment in Ingwavuma community

**DOI:** 10.3389/fpubh.2023.1050589

**Published:** 2023-06-02

**Authors:** Zinhle Mthembu, Moses Chimbari

**Affiliations:** ^1^Department of Public Health Medicine, College of Health Sciences, University of KwaZulu-Natal, Durban, South Africa; ^2^Department of Anthropology and Development Studies, Faculty of Humanities and Social Sciences, University of Zululand, KwaDlangezwa, South Africa

**Keywords:** ecohealth, involvement, communities, community engagement, health research

## Abstract

**Introduction:**

The goal of community involvement in health research is to improve a community’s ability to address its own health needs while ensuring that researchers understand and consider the community’s priorities. Recent data show that socio-economic and environmental challenges continue to be a barrier to informing, consulting, involving and empowering communities in community-based health research beneficial to them. The aim of this study was to assess the extent to which the Ingwavuma community in KwaZulu-Natal Province, in rural South Africa, was informed, consulted, involved and empowered about two research projects conducted between 2014 and 2021.

**Methods:**

The study used the modified random-route procedure to administer a standardized questionnaire to 339 household heads selected randomly. The questionnaires were administered face-to-face. The sample size was estimated using the Yamane sample size generating formula. Chi-square tests were performed to assess associations between demographic variables (age, gender, education, village) and respondents’ knowledge and information of the projects, Malaria and Bilharzia in Southern Africa and Tackling Infections to Benefit Africa as well as their participation.

**Results:**

The communities were generally well-informed about the health projects that were being carried out. Fewer than half of those who had heard about the projects had directly participated in them. The majority had been tested for one or more diseases and conditions, mostly high blood pressure, diabetes, and schistosomiasis, and had participated in a community feedback group; many had given their children’s permission to be tested for schistosomiasis or to participate in project research activities. Others participated in public awareness campaigns and surveys. There was some evidence of a consultation process in the form of public consultation discussed in the projects, and not much discussion on empowerment.

**Discussion:**

The findings demonstrate that researchers’ CE approach was adaptable as communities were largely educated, involved, and subsequently empowered though without much consultation and that researchers had provided a space for sharing responsibilities in all engagement process decision-making. For the empowerment of the community, projects should take into account the intrapersonal and personal aspects affecting the community’s capacity to effectively benefit from the information, consultation, involvement, and empowerment procedures.

## 1. Introduction

Community engagement (CE) in health research aims to strengthen a community’s ability to tackle its own health problems while drawing the attention of researchers to known health challenges in the community. Communities in which community health research is conducted must perceive the research process as authentic and credible (1). However, power imbalances between researchers and the participants (communities) that result in community members not always having a voice in the decision-making process (2) sometimes lead to the withdrawal of communities from studies due to mistrust and suspicion (3). Community trust is significantly associated with community engagement and if a community lacks trust, it may decide to disengage (4). Strong evidence found poverty and unemployment in remote rural communities influence how research participants misinterpret outside researchers as potential sources of various material benefits. Many studies have reported that there is little guidance on how to assess the CE processes, the outcomes and the impact on communities, which should lead to community empowerment (5–7). CE in community health research refers to efforts that promote the exchange of information, ideas and resources between community members and researchers. It is a collaborative co-governance of research including researchers and people affected by issues under investigation or in positions to act on research findings, such as end-users including intervention participants, health managers, and policymakers (8). Researchers can acquire knowledge and trust, but they may not fully appreciate the true community health status to adequately address pertinent research questions. On the other hand, some communities may not always trust the intentions of researchers, or the methods used in the research (9, 10). Similarly, ordinary community members may have limited research skills, knowledge and training, and may not fully appreciate the complexities of research methodology and theories (11). Therefore, while health researchers share their health expertise, services, and other resources with the community as part of community engagement in the research process, the community may provide crucial local knowledge and experience that can greatly help direct the efforts of health research projects/programs. It is important to engage communities in all research processes so that they fully benefit from the research. This implies that the concept of CE is critical in community research and the inclusion of communities in the research process from inception can improve the way research is planned, carried out and used (12).

CE improves health outcomes by increasing the cultural and logistical adaptation of community-based research projects to their settings, promoting community empowerment, and facilitating the translation of research-generated health knowledge into practice (13). In addition, CE can help to uncover the social, political, and economic contexts that underpin both facilitators and barriers to knowledge and resources needed for health (14), especially when the research process is co-governed with end users. Although community engagement is considered important in health research, its implementation is still understudied (15). CE can be challenging as it requires effort, capacity, investment of time as well as money (16) and the researched community may be indispensable regarding the methods and execution of the project (17). Therefore, dialogues between community members and researchers with different levels of involvement, decision-making and control between community and health researchers can overcome these challenges (18). Consequently, researchers must approach communities as research partners, with community members and leaders’ participation viewed as critical for acceptability and success of a research project/program. The challenges are amplified when a particular health issue or research question is not prominent in the consciousness of the targeted community.

In community health research settings, investigators and their teams must inform, consult, involve and empower the community about the objectives, rationale and benefits of research projects for the community. However, little is often known about the extent to which they are informed and/or educated about health research projects in their localities/communities. Without a clear assessment and understanding of the extent of information and communication communities received from research project teams, researchers are likely to fail in their attempts to involve community members in research collaborations. Establishing a research partnership without effective communication and information can lead to decisions and actions that further violate the trust of the community. Distrust not only affects the immediate research relationship, and, in turn, the validity of the data collected, but also has a profound impact on the future willingness of the affected populations to engage in the research enterprise. In this article, we present findings on the extent to which a local community was informed, consulted, involved and empowered about research projects and related activities in their locality. It is based on two community based projects; Malaria and Bilharzia in Southern Africa (MABISA) and Tackling Infections to Benefit Africa-South Africa (TIBA-SA) (19), carried out between 2014 and 2021 in the Ingwavuma area of KwaZulu-Natal Province in rural South Africa.

## 2. Materials and methods

### 2.1. Study setting and MABISA/TIBA-SA project overview

**Figure 1:**
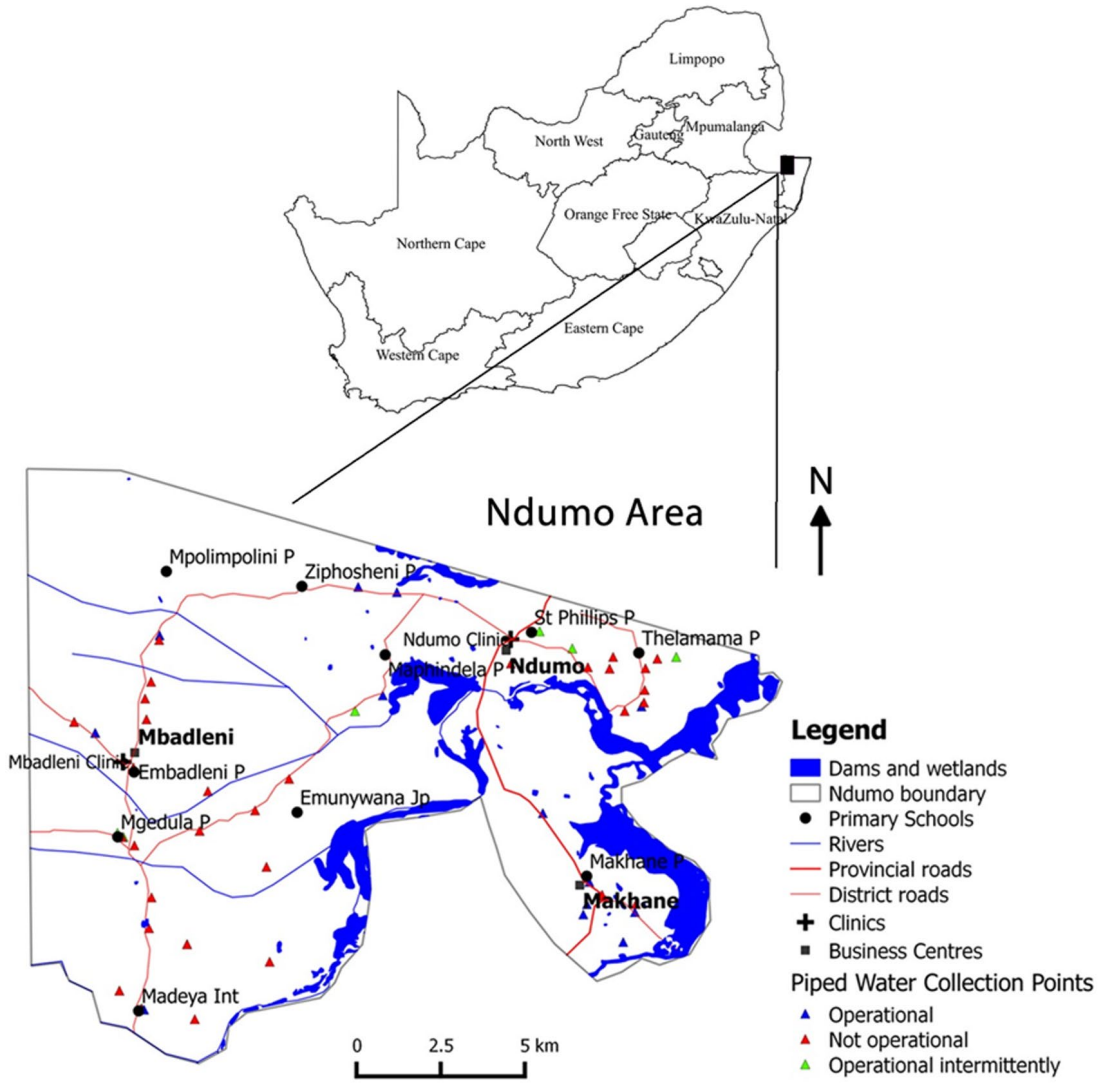
Map of Ingwavuma areas, uMkhanyakude district, Jozini Municipality, KwaZulu-Natal, South Africa.

This study was conducted in Ingwavuma, an underdeveloped area in the uMkhanyakude district, KwaZulu-Natal province, South Africa (20). The area lies on the north-east border with Mozambique and Swaziland and is adjacent to the Ndumo game reserve (Figure 1). A permanent river, the Pongola River, flows through it. The Pongola has distributaries that start from within the mountains that border Swaziland, one of which is the Ingwavuma River. There is very little infrastructure in this area; the road network is still being developed and much of the area is accessible through gravel roads. Schools are sparsely distributed throughout the villages and offer minimum utilities with most of them having no tap water. Due to the dry weather conditions in the region, agricultural activities and other related economic activities are limited. Apart from an irrigation system that draws water from the Pongola Dam, which is more than 35 km away, there is no other irrigation system. The town of Ingwavuma is located in a low-lying area, characterised by hot temperatures, stagnant and slowly moving water bodies. These geographical conditions make the region a hotspot for schistosomiasis and malaria. Individuals in these areas experience extreme poverty and low levels of education. These factors indicate the need to involve the community in health education in a robust and inclusive way.

The MABISA (Malaria and Bilharzia in southern Africa) and TIBA-SA (Tackling Infections to Benefit Africa-South Africa) research projects were initiated in 2013 and 2017 respectively, upon realization that social and environmental determinants of health have a major influence on the epidemiology of vector-borne diseases (VBDs) and that the influence is exacerbated by climate change (19). The Ingwavuma area faces different socio-economic and environmental challenges, which offer opportunities to investigate the impact of these factors on VBDs. These community-based health research projects were designed to address the impact of social-environmental determinants and climate change on two VBDs, malaria and schistosomiasis. The projects focused on the ecologies and water systems of dry land (rivers, lakes, rain-fed systems, irrigation systems) within dry land in order to develop adaptation strategies to reduce vulnerability to these diseases in population health. TIBA-SA had components of BP and Diabetes project. Throughout the projects, the study team relied on the input of members of the community to guide the nature and structure of interventions.

The paper is based on work in a larger project, the KwaZulu-Natal Ecohealth Program (KEP) which uses a participatory action research methodology. A governance structure and an operational strategy that involves the community to ensure that the community fully participated in MABISA/TIBA-SA projects was established during the first phase (Informing) of CE. A 12-member Community Advisory Board (CAB) comprising of one headman (induna), two community leaders, three school board members, three community care givers and three ordinary community members was established at the inception of the MABISA project and is functional to date. The headmen (izinduna) are the elected gatekeepers with authority over villages and are accountable to the chiefs, the tribal council and their community (21). The informing of the community in the MABISA project was through the community liaison officer (CLO), who was referred to the MABISA project by the Provincial Health District, as he had previously worked for other NGO projects in the area. The CLO then linked the principal investigators with the leaders of the community to introduce the ideas of the project. The project principal investigators visited the community with a prepared detailed study document to engage with the community. In this methodology, it is important to note that the researchers were the ones who came up with the idea for the project, found a way to get the community involved by informing them about the project, and engaged community members to have input on the design, methodology, and execution of the project. The community meeting was organised by the induna and activities for participatory rural appraisal (PRA) (origins of PRA) were conducted to identify issues that were to be researched. This method of engagement gives a community access to accurate and objective information that will assist them in better understanding the project proposed as well as the potential solutions.

The project field operations were carried out by researchers and CRAs as they were trained to attain the required skills for the fieldwork. The presence of the CAB and CRAs has been instrumental in promoting the concept of community change makers for prevention and control of vector borne diseases including zoonosis. Decisions concerning survey design and implementation, as well as techniques to collecting anthropometric and biochemical data, were influenced by community leaders and community members employed as staff (CRAs). The initiatives in the TIBA-SA projects are designed to have an influence on the society that is affected by health issues; they take an Ecohealth approach with a focus on community participation. The projects were founded on the idea that academics working with the Ingwavuma community would be able to change health behaviour, collect information, and increase understanding of infectious illnesses including schistosomiasis and malaria. The project produced good results which were largely disseminated through various means, workshops, local radio station and journal publications (much less for communities and government agencies). As part of the uptake activities, we had school children drama competitions focusing on malaria and schistosomiasis. We have realized that edutainment is an effective way of disseminating information to communities and that uptake is likely when the information is naturally assimilated through music, dance, poetry and drama. We used a group called Ubuciko, the Art to provide edutainment. During MABISA project they performed during PRA meetings and the response from the community was overwhelming in terms of information dissemination. This method of information dissemination resonance well with the culture of communities we worked in. In addition, a community feedback meeting was used as a platform to disseminate findings of the project to all stakeholders. Because of the capacitation of CRAs we made and involvement of Department of Health personnel we believe that the project ideas will be sustained in the community and relevant government departments.

#### 2.1.1. The community engagement Vancouver coastal health framework

The study applied the Community Engagement Vancouver Coastal Health framework^1^ which involves five CE components. The stages include (1) informing the community, (2) consulting the community, (3) involving the community, (4) collaborating with the community, and (5) empowering the community and are summarized in Table 1. This paper focuses on the Informing, Consultation, Involving and Empowerment phases. Although there are different methods and frameworks for community engagement, this framework, often quoted in many studies (22–26) was suitable for this study. We wanted to engage the community across the full spectrum of participation levels ranging from informing, consulting and involving to collaborating and empowering. The Vancouver framework outlines community engagement as public participation and is based on the principle that people have the right to participate in the decision-making processes that affect them and that everyone has a say when it comes to their health care (27). This framework was adapted from Sherry Arnsteins’s theory of Ladder of Citizen Participation which is one of the most widely referenced and influential models in the field of democratic public participation (28). Arnstein’s theory discusses about eight levels of participation arranged in a ladder pattern with each rung corresponding to the extent of citizens’ power in determining the end product. The bottom rungs of the ladder are, first (*Manipulation*), and second (*Therapy*), which describes “non-participation” real purpose here is not to give individuals a voice in planning or executing initiatives, but rather to provide those in positions of authority the opportunity to “educate” or “cure” the people who are involved (28). In the third (*Informing*) and fourth (*Consultation*) rungs, “tokenism” increases to the point that the have-nots can finally be heard and their voices heard. Rung fifth (*Placation*) is simply a higher-level tokenism because the ground rules allow have-nots to advise, but retain for the powerholders the continued right to decide. Rung six (*Partnership*) enables them to negotiate and engage in trade-offs with traditional powerholders. At the top most rungs, seven (*Delegated Power*) and eight (*Citizen Control*) have-not citizens obtain the majority of decision-making seats, or full managerial power (28). For local leaders, organizers, and facilitators who want to understand foundational theories of public engagement and participation, and the ways in which empowered public institutions and officials deny power to citizens, Arnstein’s theory was also essential for this particular study aimed to assess the extent to which the community, in rural South Africa, was informed, consulted, involved and empowered about two research projects (MABISA and TIBA-SA).

**Table 1:** Five components of community engagement.

Inform	Consult	Involve	Collaborate	Empower
**Objective**	**Objective**	**Objective**	**Objective**	**Objective**
To provide community with balanced and objective information to assist them in understanding the problem, alternatives, or solutions.	To obtain community feedback on analysis, alternatives, or decisions.	To work directly with the community throughout the entire process to ensure that community and organizational concerns are consistently understood and considered.	To partner with the community in each aspect of the decision including the development of alternatives and the identification of the preferred solution.	To place final decision-making in the hands of the community
**Promise to the community**	**Promise to the community**	**Promise to the community**	**Promise to the community**	**Promise to the community**
We will keep you informed.	We will keep you informed, listen to and acknowledge your concerns, and provide feedback on how community input influenced the decision.	We will work with you to ensure your concerns and issues are directly reflected in alternatives developed and provide feedback on how community input influenced the decision.	We will look to you for direct advice and innovation in formulating solutions and incorporate your advice and recommendations into the decisions to the maximum extent possible.	To place final decision making in the hands of the community.

### 2.2. Study participants and data collection

The study was carried out between November 2019 and November 2021. The study used the modified random-route procedure (29) to administer a standardized questionnaire to 339 household heads selected randomly. The sample size was estimated using the Yamane sample size generating formula (30). The modified random route procedure involved dropping interviewers at different locations within the designated geographical area and allowing them to choose a starting point and direction for the selection of households. Since this method is employed when there is not a complete list of households, it aims to produce equal selection probabilities so that each household has an equal chance of being included in the sample (31). Questions were arranged in a logical sequence and uploaded to KoboCollect (32), an online open source platform for data collection and analysis. Questionnaires were administered face-to-face.

The questionnaire was designed in English and translated into the study area local language, isiZulu. Community Research Assistants (CRAs) who administered the questionnaire received intensive training over 2 days. In order to ensure uniform understanding and evaluation of data collection, the instrument was pre-tested in one of the villages in the area, with similar socio-demographic and cultural characteristics to the study area. The village where pre-testing was done was excluded from the main study. Additional modifications to the tool were done based on the results from the pre-testing. The questionnaire included questions on demographics, such as age, gender, and the level of education of the household heads. In order to determine how informed the community was about research projects in their locality, respondents were asked to name any health research project they remembered to have been conducted in their community in the past 7 years during which the two projects were undertaken in the community. Those who had lived in the research area for over 10 years may have had rich information. They were also asked whether they had ever heard of MABISA/TIBA-SA research projects, among other questions. Items were designed to be closed ended, but an option for additional open-ended responses was included for most of the questions.

### 2.3. Data analysis

Data were analysed using descriptive statistics specifically frequencies and percentages. Chi-square tests of associations were done to assess associations between demographic variables (age, gender, education, village) and respondents’ knowledge and information of the MABISA/TIBA-SA projects, their involvement as well as empowerment. Further Chi-square tests were done to assess the association between participating in the study and knowledge about its aims, activities, researchers, sites as well as whether respondents believed they had benefitted from the projects. Cramer’s V tests were applied to all statistically significant Chi-square tests to measure the strengths of associations while descriptive contingency tables were used to identify relationships within the associations with a V of 0 indicating no relationship and a V of 1 showing the strongest possible association between tested variables (33). A probability value of 0.05 was used in both the Chi-square and Cramer’s V tests. The general view behind the tests was that an informed, consulted, involved and empowered audience would exhibit statistically significant results that showed strong associations between project participation and knowledge of the projects’ aims, activities, researchers and research sites. Also, they would show a strong association between project participation and benefits.

### 2.4. Ethical considerations

Ethical approval was obtained from the University of KwaZulu-Natal (UKZN) Institutional Ethics Board, Humanities and Social Sciences Research Ethics Committee (HSSREC), Protocol reference number: (HSSREC/0001650/2020). All participants gave informed consent to participate in the study.

## 3. Results

### 3.1. Participants demographics

Table 2 below shows that respondents’ demographic information. The study used a sample of 339 respondents from five villages in the Ingwavuma Community.

**Table 2:** Study participant demographics.

Description	Participants	Frequency	Percent
Gender	Males	92	27.3
	Females	245	72.7
Age (Years)	<25	89	26.3
	26–35	91	26.9
	36–45	57	16.9
	46–55	45	13.3
	56–65	35	10.4
	66–75	11	3.3
	>75	10	3.0
Village	Ndumo	107	31.7
	Mbadleni	73	21.6
	Mgedula	49	14.5
	Madeya	23	6.8
	Makhane	86	25.4
Highest Education level	No formal education	60	17.8
	Primary	83	24.6
	Secondary	164	48.5
	College level	9	2.7
	Above college level	3	0.9
	Other	19	5.6

Of the five villages, 107 (31%) respondents came from Ndumo followed by 85 (25%) from Makhane (see Table 2). The majority of respondents (88%) had stayed in the study area for more than 10 years which could mean that they had rich information about the community projects and what happens in their community. More than 26.9% of respondents were aged 35 years and below, while those above 66 to 75 of age were 3.3%. Further, the results show that more than two-thirds (72%) of the households surveyed are female-headed and that most (over 90%) have secondary education and less as their highest level of education.

As indicated in Table 3, out of 338 respondents, 177 (52.4%) had heard about the MABISA/TIBA projects while 161 (47.6%) had not. Of the 177, 41.8% participated in the projects. Less than half of the respondents who had heard about the projects were involved as participants. Of the 177 respondents who said they had heard about the MABISA/TIBA projects, 64.4% stated that they were familiar with the projects on Schistosomiasis, 20.3% with the Malaria project while 18.6% said they had forgotten about the project they had heard about. Respectively, 10.7, 7.3 and 4.5% of the respondents who knew about and who had heard about the MABISA/TIBA projects knew about the BP, Diabetes and infectious diseases/diseases projects. The participants were therefore exposed to information about different projects with some having no information about running projects. A considerable number reported to be uninformed or having forgotten about some projects. Among the 177 respondents who had heard about the projects, 43.5% had heard about these from CCGs, 28.2% from schools, 15.3% from family/neighbours and 13.6% from community meetings. Also, 2.8% had heard about these from their traditional leadership and another 2.8% from television/radio. CCGs and schools were therefore the commonest sources of MABISA/TIBA projects information.

**Table 3:** Information on health research projects and involvement.

	Responses	Frequency	Percent
C2 Have you ever heard of MABISA/TIBA projects?	No	161	47.6%
	Yes	177	52.4%
	Total	338	100.0%
Did you participate in the project?	No	103	58.2%
	Yes	74	41.8%
	Total	177	100.0%
C3 Mention MABISA/TIBA projects that you know about:	Schistosomiasis	114	64.4%
	Malaria	36	20.3%
	Do not know/have forgotten	33	18.6%
	BP	19	10.7%
	Diabetes	13	7.3%
	Infectious diseases/diseases	8	4.5%
	HIV	1	0.6%
Where did you hear about these projects?	CCG	77	43.5%
	School	50	28.2%
	Family/neighbours	27	15.3%
	Community meeting	24	13.6%
	Other	16	9.0%
	Traditional leadership	5	2.8%
	Television/radio	5	2.8%

### 3.2. Participation and benefits

The respondents were asked if they took part in any of the projects and what they had learnt from them. Table 4 summarises their responses.

**Table 4:** Participation and benefits.

	Question/Statement	Frequency	Percent
Did you participate in the projects? if yes how?	Tested for diseases and conditions	35	46.1%
	Survey respondent	5	6.6%
	Consented for children to participate	9	11.8%
	Community feedback group	19	25.7%
	Training and awareness recipient	8	10.5%
What did you learn or understand from the projects that are being done in your community?	Schistosomiasis	53	68.8%
	Personal health and hygiene	34	44.2%
	Malaria	23	29.9%
	Infectious diseases in general	16	20.1%
	I have forgotten	10	13.0%
	Diabetes	8	10.4%
	Importance of visiting healthcare facilities	6	7.8%
	BP	6	7.8%
	Nothing	5	6.5%
	HIV/STDs	5	6.5%
	Importance of taking medication	4	5.2%
	Cancer	4	5.2%

Out of 76 respondents, 46.1% had been tested for one or more diseases and conditions, mostly BP, Diabetes and Schistosomiasis. Also, 25.7% had participated as part of a community feedback group and 11.8% had participated by consenting for their children to be tested or to take part in the projects’ research activities. 10.5% participated in awareness campaigns and 6.6% participated as survey respondents.

The above Table 4 focuses on 76 respondents who responded “Yes” to the question *What did you learn or understand from the projects that are being done in your community?* Out of the 77, most of the respondents (68.8) learnt about Schistosomiasis, 44.2% about personal health and hygiene, 29.9% about Malaria and 20.1% about infectious diseases in general. Of these, 13% reported to have forgotten what they learnt while 6.5% said they learnt nothing from the projects. The majority had therefore benefitted through learning about Schistosomiasis. A minority had also learnt about Malaria and infectious diseases.

### 3.3. Associations between informing, involvement and participation

Table 5 analyses data from the 177 respondents who had participated in the projects’ activities focusing on associations between participation, informing and consultation.

**Table 5:** Informing and involvement and participation.

Statement/Question		C16 Did you participate in the project?			*X*^2^		Cramer’s V	
	Response	No	Yes	Total	Stat	Sig.	Stat	Sig.
C5 Do you know the research activities conducted by MABISA/TIBA projects?	No	61	22	81				
	Yes	42	52	94				
	Total	**103**	**74**	177				
C7 Do you know the project’s research sites?	No	99	54	153	19.68	0.00	0.33	0.00
	Yes	4	20	24				
	Total	103	74	177				
C8 Can you list the aims of the research project?	No	101	55	156	23.2	0.00	3.62	0.00
	Yes	2	19	21				
	Total	103	74	177				
C12 Have you met the MABISA/TIBA researchers?	No	74	5	79	73.8	0.00	0.65	0.00
	Yes	29	69	98				
	Total	103	74	177				
C14 Can you list the names of the research team?	No	86	41	127	16.8	0.00	0.31	0.00
	Yes	17	33	50				
	Total	103	74	177				
C18 Did you sign the consent form before you participated in the research project?	No	103	3	106	165.02	0.00	0.97	0.00
	Yes	0	71	71				
	Total	103	74	177				
C20 Did you get enough background information about the project from the consent form?	No	103	3	106	165.02	0.00	0.97	0.00
	Yes	0	71	71				
	Total	103	74	177				
Benefitted	No	51	48	99	4.11	0.04	0.15	0.04
	Yes	52	26	78				
	Total	103	74	177				

The bold values are the total of respondents who participated in the projects.

Out of 74 respondents who participated in the projects 52 (70.3%) of the respondents knew about the research activities conducted by MABISA/TIBA projects. The remaining 22 (29.7%), despite taking part, were not aware of the organisation’s projects. Also out of the 74, only 20 (27%) were familiar with the projects’ research sites. The remaining respondents had no idea about these projects. In the same group, 19 out of 74 (25.7%) participants knew about the projects’ research aims while the rest did not. The majority of the respondents (69 out of 74) or 93.2% had, however, met directly with the MABISA/TIBA researchers while only 5 had not. Also, 33 out of 74 (44.6%) knew the researchers’ names while the rest did not suggest that despite this contact, some respondents remained poorly informed about the research projects’ aims and sites. Almost all the respondents who had participated in the MABISA/TIBA projects stated that they had signed a consent form, and the same number also affirmed that these consent forms had enough background information about the project. Finally, only 26 out of 74 (35.1%) respondents said they had benefitted from the projects while 48 (64.9%) said they had not.

In the above crosstabulations, statistically significant *X*^2^ confirms the association between participating in the projects and knowing about them specifically the activities conducted in them (*X*^2^(1) = 15, *p* = 0.00); participation and knowledge of research sites (*X*^2^(1) = 19.68, *p* = 0.00), aims (*X*^2^(1) = 23.2, *p* = 0.00) and researchers involved (*X*^2^(1) = 16.8, *p* = 0.00). In all these associations, Cramer’s V ranged from 0.15 on the benefits to 0.97 on consent. Besides the low size effect (low Cramer’s V) on the benefits of participation, moderately strong to very strong associations were recorded these being highest on consent (Cramer’s V = 0.97, *p* = 0.00).

However, the results point to a weak association between participation and benefitting from the projects (*X*^2^(1) = 4.11, *p* = 0.00) further supported by a Cramer’s V of 0.15. Ironically, more respondents (50.5% or 52 out of 103) reported to have benefited from the projects without participating compared to 35.1% (26 out of 74) who benefitted from direct participation. The project, therefore, had an impact beyond those who were directly reached out to as information about projects also filtered to those who did not directly participate. The data above also highlights some inconsistencies that suggest limited information on the part of the respondents. Specifically, 71 out of 74 reported that they had received and signed consent forms that provided them with research projects’ adequate background. This is despite 19 out of 74 stating that they did not know of the projects’ research aims. The above data highlights the following patterns: Involvement without critical full information (aims, activities, identities, sites of the projects); Poor understanding of the research consent process among the participants and consequentially low benefits from involvement/participation.

### 3.4. The association between information and demographic groups

There were no statistically significant associations between the question – *Can you list the aims of the research project?* and the variables gender, age, level of education, village and the number of years one had stayed in the surveyed community. The same applied to the association between the question *Did you participate in the projects?* And the above variables.

There was also no statistically significant association between benefiting from the projects and the variables age, level of education, village and the number of years one had stayed in the surveyed community. Persons of different genders however benefitted differently from the projects as shown by statistically significant *X*^2.^(see Table 6).

**Table 6:** Association between gender and benefits from the projects.

		*X*^2^		Cramer’s V	
	Stat	Sig.	Stat	Sig.
	Benefitted						
	No	Yes	Total				
Female	80	53	133				
Male	19	25	44				
Total	99	78	177				

Females benefitted less (53 out of 133 or 39.8%) than males (56.8%). A Cramer’s V of 0.148, however, indicates that this association was not very strong. The data in this subsection demonstrates an inclusive approach to community engagement by indicating that information and involvement in the projects were not centred towards specific demographic groups.

## 4. Discussion

**Figure 2:**
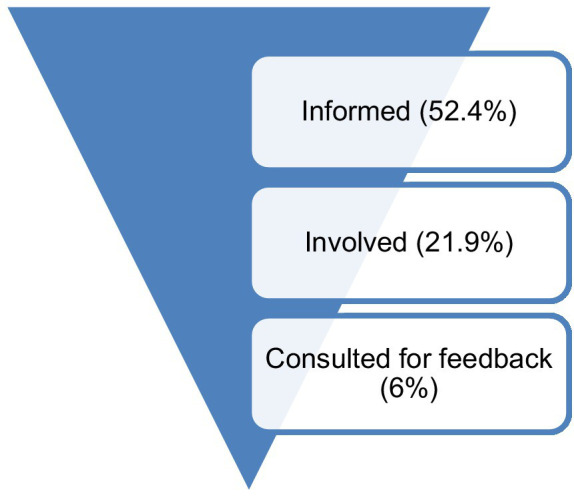
Summary (informed, involved and consulted participants) – Researchers (Source: Author, 2022).

The findings from this study show varying dynamics in the respondent’s levels of information and involvement in MABISA/TIBA projects. From the sample, 52.4% of the 339 respondents indicated that they were informed of the projects, 21.9% participated in them and 6% were consulted for feedback as illustrated in Figure 2.

More respondents benefitted from the projects than those who participated. Nonetheless, the data showed an inverted pyramid pattern where fewer respondents progress to the next advanced participation phases. A 52.4% project awareness rate, however, could be justifiable considering the rural nature of the communities involved. The conversion from being an informed person to a participant highlights potential challenges in getting communities involved in the projects. Such limited interest to participate highlights engagement challenges that include, among other things, feeling marginalized, and failure to identify with research and project purposes and methods among others (34).

### 4.1. Informing

The study identified two major levels of public information involved in the projects. The first was to inform whole communities of the projects’ existence. The second level involved informing part of the communities that chose to participate in the research. The data shows that the five communities that took part in the research exhibited poor levels of information about the projects on both levels. Approximately half of the respondents had never heard of the projects.

Among those who had heard about the projects and chose to participate, there were critical information asymmetries between the researchers and the participants on what the project was about, where it was based, who their researcher was and what activities it involved (35). These are considered key aspects that define research, yet they remain unknown to respondents. One of the major activities and tools used to inform communities about research is the consenting process, regularly done through the handing over and signing of a consent form. Almost all participants went through this. While that process provided all the required project background it failed to have the desired impact on the researched communities. Lack of knowledge cannot be blamed for the poor understanding of the projects’ aims, as there were no statistically significant differences in this regard.

The consent issues raised above highlight possibilities of poor understanding of the consenting process among research respondents (36). The University of California, San Diego Brief Assessment of Capacity to Consent Questionnaire (UBACC) is one of the reliable and validated tool that can be used routinely to assess individuals’ capacity to consent to a research protocol and improve understanding of research study through iterative learning (37). The UBACC is designed to assist researchers in identifying study participants who require a more comprehensive decisional capacity assessment and/or remedial efforts prior to enrolment (37). If individuals are assessed using the UBACC, it provides more certainty that they are informed about what they are enrolling for. This is particularly important given some risks of health research. Research respondents may agree to participate in a study without knowing or rigorously looking at its pertinent details. Procedural weaknesses particularly the handing over of a consent form together with the questionnaire for immediate filling can also deny respondents the chance to fully grasp what they are consenting to Ref. (36). Relatable findings were also made in the study on how rural community respondents fail to recall the contents of consent forms (38). In the study that was conducted in rural KwaZulu-Natal, some respondents had participated in projects under the belief that they would materially benefit from them. The process of getting meaningful consent to clinical research participation may be hampered by therapeutic misconception, which occurs when research subjects fail to appreciate the distinction between the imperatives of clinical research and ordinary treatment, and therefore inaccurately attributes therapeutic intent to research procedures (39). A study demonstrated that therapeutic misconception occurs in the South African oncology research setting and has the potential to lead to underestimating of the risks of a Phase 3 clinical trial. Therefore, it is vital to emphasise the experimental nature of a clinical trial throughout the consent process in order to overcome therapeutic misconceptions in oncology research (40). Another South African study on the ethical challenges in obtaining informed consent for genomic research in general and the implications of recruiting healthy controls for genomic research in particular discovered that diagnostic and therapeutic misconceptions were the main challenges (41). In terms of informed participation, this creates a risk of a sample that is not fully involved in a study due to information asymmetries between the researcher and the research targets (42). Such targets, reveal vulnerabilities that the researchers must consider during the research process (43).

The linkage of consent challenges to the quality of participation has previously been explored (44). Passive participation occurs when respondents consent to research they may not fully understand, either lack of knowledge or out of being poorly oriented by the researcher. Passive participation is also common when passive consent approaches are applied in research (45). In this study, the enigma is that the respondents asserted that they participated voluntarily and were fully informed of the research background, yet they were unable to identify the researchers, the research aims and sites. Therefore, before consenting to research studies, potential participants should be informed about research objectives, procedures, and benefits and risks to minimise diagnostic and therapeutic misconceptions (41). Before enrolling participants in research studies, researchers must assess their familiarity of scientific jargon and concepts. Research team recruiting potential study participants must be innovative and ethical during the process. Researchers must be able to adapt consent processes to the recruitment setting to help potential study participants make informed decisions (39–41).

### 4.2. Consultation

We found some evidence of consultative processes. Twenty-five-point 7 percent (25.7%) of the respondents who participated in the projects were consulted for feedback relating to the projects. Further to that, the largest percentage of respondents had heard about the projects from community caregivers. Community meetings were also another form of public consultation discussed in the projects. However, the overall data suggests that such consultative practices, despite being procedural might not have resulted in quality information output as the consulted groups mostly indicated lack of knowledge about the projects (aims, identity, activities, researchers and sites). Furthermore, the respondents did not list consultative processes as an ongoing research activity like the surveys they listed. This also suggests that the consultation processes were for post-evaluation processes (46) posit that community consultations in research projects were a basis for the creation of mutually beneficial and more productive engagements between researchers and communities. Such consultations span include methodological and content processes that can enhance the attainment of project goals (46). Consultation is a form of involvement that supports the co-creation of projects and wider participation of marginal and disadvantaged groups and communities that may otherwise be excluded from research institutions (34). This view applies to the five remote communities studied. Consultative processes help to clear up such misconceptions, thereby improving the quality of health research interventions. Poverty and unemployment in remote rural communities have been found to have a strong influence on how research participants misinterpret outside researchers as potential sources of various material benefits (38). Consultative processes iron out such misconceptions enhancing the quality of health research interventions.

### 4.3. Involvement

Defining involvement from the Community Engagement Vancouver Coastal Health framework there is evidence of limited public involvement from the results. This is highlighted in the majority of the respondents’ failure to identify the project, specifically, its project sites and researchers. As expected, community engagement efforts generally result in the availing of such information to the research subjects. While the majority of the respondents who participated in the study acknowledged meeting the researchers, the failure to identify them suggests limited engagement. Limited engagement disempowers communities and limits their contribution, as well as benefits, from health projects (46).

### 4.4. Collaboration and empowerment

In the study, there is not much discussion on research collaboration between the researchers and the communities. As highlighted, community members were primarily the participants for surveys, beneficiaries of awareness programmes, and test subjects for medical examinations and screenings. A small fraction did give its feedback to the researchers as discussed earlier. Thus, according to the Community Engagement Vancouver Coastal Health framework, the research projects may be discussed as reaching the informed and involved stages. The advantages of community empowerment in research include more active and wider participation that supports desired behavioural change (47). Such benefits might have been missed by the research.

**Figure 3:**
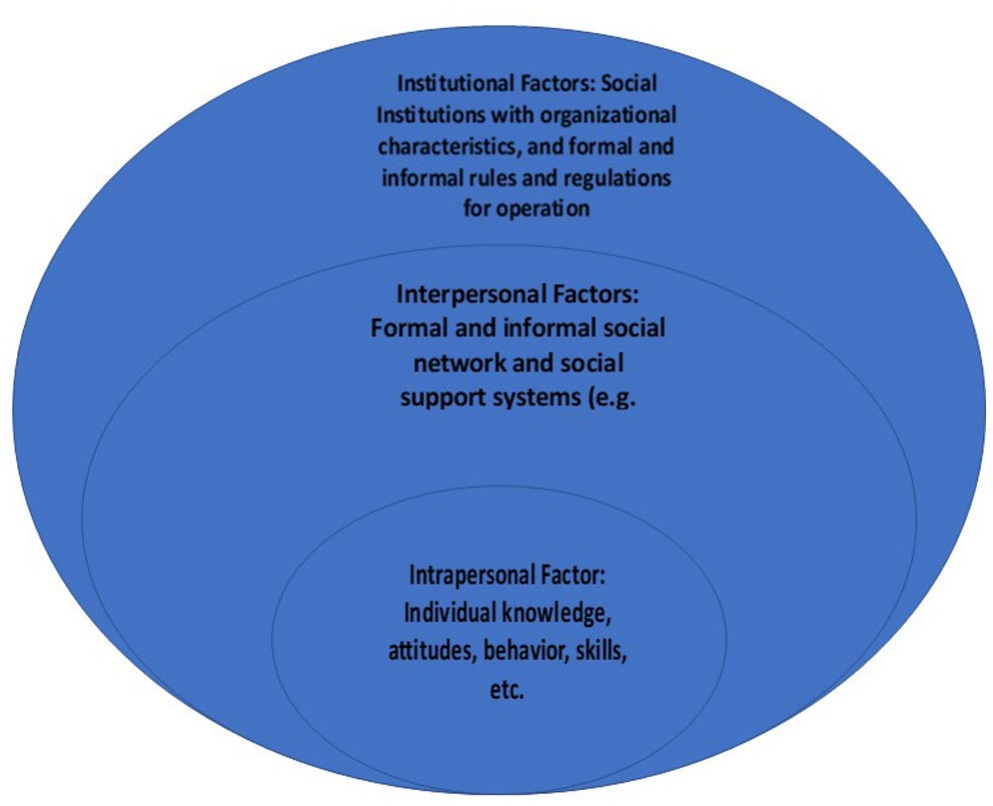
Barriers and facilitators of participation/involvement (48).

Tests of associations attempted to find relationships between information and involvement (participation) and demographic data. As indicated in Figure 3, the first dimension affecting participation consists of intrapersonal factors (48).

From the projects, the pattern of being poorly informed and less keen on participation was common among respondents of different ages, genders and levels of education. This points to the limited dominance of intrapersonal factors in the participation processes (48). This suggests that interpersonal and institutional factors may be more responsible for low information and low participation in the projects. At the same time, however, some respondents did signify that despite being engaged and informed, they had forgotten what the projects were all about, pointing to some intrapersonal limitations affecting information and involvement as well. In the conceptual model presented above in Figure 3, this could indicate knowledge retention and cognitive issues or even attitudinal issues that affect participation levels (48).

The same study by Bay et al. (48) also points to the importance of formal and informal social networks in research participation – similar to this study. Participation through social networks was mostly evident in the number of respondents who benefitted from the research projects without directly being part of them – mainly through information flows. Parents indicated benefiting from the projects through their schoolchildren whom they consented to participate. Other respondents noted that they only knew about the projects from neighbours and family. This shows a strong interpersonal component in participation.

### 4.5. The study’s proposed framework

We propose using a hybrid framework the Community Engagement Vancouver Coastal Health framework and Bay et al.’s (48) conceptual framework on factors that affected individual and community participation in health projects (see Table 7) below.

**Table 7:** The engagement-empowerment framework (Researchers).

		Levels of engagement		
		**Intrapersonal**	**Interpersonal**	**Institutional**
Levels of participation and decision-making	**Inform**	Individual characteristics and understanding	Group characteristics and understanding	Institutional systems and processes to support intrapersonal and interpersonal information
	**Consult**	Consult individuals based on their nature and understanding of the project	Consult unique groups and niches based on their nature and understanding of the project	Institutional systems and processes to support individual and group consultation
	**Involve**	Involve individuals based on their nature and understanding of the project and consultation outcomes	Involve groups based on their nature and understanding of the project and consultation outcomes	Institutional systems and processes to support individual and group consultation
	**Empower**	Empower individuals to effectively make decisions about the project processes based on their personal capacities	Empower groups to effectively make decisions about the project processes based on identified group characteristics	Develop institutional systems and processes to support individual and group decision-making and control of projects

The framework highlights a need to engage communities more meaningfully in research through effectively informing, consulting, involving and empowering processes. Our study found that the communities were mostly informed and involved but were not fully consulted and not empowered. To empower communities, projects should consider the intrapersonal and personal factors affecting community capacity to fully benefit from the information, consultation, involvement, and empowerment processes. In our study, poor understanding of the project was suggested to be a result of both intrapersonal issues – for example, respondents stated that they were involved in the project but did not understand or had forgotten what it was about. Intrapersonal factors came out as important in the dissemination of information about the project. Health research institutions had the role to develop research methods and processes that took into consideration the intrapersonal and interpersonal characteristics of the communities involved.

## 5. Limitations

A CE strategy that fully engages the community is limited by community research literacy levels, time, and resources, but creates an environment conducive for research. The time lapse between when participants participated in the MABISA and TIBA-SA research projects have impacted their collection of details such as researcher’s names and particulars about the study. This paper currently does not address how the community was “empowered, “because measuring community empowerment may be difficult. The CE framework that forms the foundation for the study is Canadian. Apart from extensive literature in CE, there is a dearth of empirical research conducted using the Community Engagement Vancouver Coastal Health framework in a South African context. Therefore, this framework may not be generalised to apply to all studies conducted in South Africa, but it can be transferrable to communities in similar contexts seeking to strengthen the process of how researchers truly engage communities. Factors that contributed to limited dissemination of findings to the community are varied including but not limited to inadequate funding to produce materials in vernacular languages, incomplete data sets for developing adaptation strategies, limited engagement with other partners like NGOs and in adequate knowledge on communities’ preferred channels for dissemination. It is important that the findings of the study are adequately and effectively disseminated within the community and are applied to reduce vulnerability of the communities to diseases.

## 6. Conclusion

Overall, the findings indicate that participants had limited knowledge of the two projects in which they had participated in, as well as an approach to the projects that was researcher-driven and community-responsive. While the researchers endeavoured to inform almost all the participants, critical information asymmetries exist in the community’s understanding of research project activities, aims, sites and identities. This is despite some respondents being asked to provide feedback on the projects. The findings also show evidence of the interaction of multi-level factors affecting effective participation (information, consultation and involvement) in community research projects. Individual-level factors affected knowledge retention while interpersonal factors played a role in information dissemination creating risks that poorly connected respondents might be left out. The institutional level – consisting of the researchers, their systems and processes exhibit challenges in informing the wider communities about the projects (noting the rural nature of the communities), weaknesses in converting community members with project knowledge into active participants, enhancing consultative processes beyond feedback mechanism systems and most importantly a questionably managed consent process where respondents appeared to consent to something they did not fully understand. Nonetheless, the communities recorded benefits that included learning and being tested for Schistosomiasis and Malaria among other things. Such benefits were also appreciated by community members who had not directly participated in the projects highlighting the project’s potential in disseminating information deeper into communities.

In addition to the framework discussed in Table 7, we recommend the following approaches: First, the projects needed to streamline consent processes to ensure that community members know the projects’ aims, identities and activities. Second, the projects needed to widen their outreach by utilising informal communication systems and social networks as information and involvement drivers. Third, the projects’ participation systems needed to consider cognitive challenges among participants through information aids that enhanced both the understanding and remembering of information disseminated to participants. Finally, the projects needed to follow more community-engaging approaches. Community empowerment through consulting participants on the methods, processes and activities to include for improving the projects has the potential to improve participant interest, knowledge and understanding of the projects.

## Data availability statement

The raw data supporting the conclusions of this article will be made available by the authors, without undue reservation.

## Ethics statement

The studies involving human participants were reviewed and approved by Humanities and Social Sciences Research Ethics Committee. The patients/participants provided their written informed consent to participate in this study.

## Author contributions

ZM led all aspects of the paper’s development from conceptualization and designing the study, data collection, analysis, and reporting. MC guided the process of manuscript writing and critically reviewed and edited all drafts of the manuscript. All authors contributed to the article and approved the submitted version.

## Funding

This study was supported by the National Research Foundation (NRF) (grant number: 116263) through my supervisor (MC) from the University of KwaZulu-Natal, College of Health Sciences.

## Conflict of interest

The authors declare that the research was conducted in the absence of any commercial or financial relationships that could be construed as a potential conflict of interest.

## Publisher’s note

All claims expressed in this article are solely those of the authors and do not necessarily represent those of their affiliated organizations, or those of the publisher, the editors and the reviewers. Any product that may be evaluated in this article, or claim that may be made by its manufacturer, is not guaranteed or endorsed by the publisher.

## Footnotes

^1^ www.vch.ca/ce
